# American

**Published:** 1896-04

**Authors:** 


### AMERICAN VETERINARY COLLEGE.

THE closing exercises were held at Chickering Hall, New York City, on March 25th. Out of a class of twenty-eight, two failed to pass the final examination successfully. A complete report will appear in the May number.

